# Simulation of Groundwater Pollutant Transport Characteristics in Mining Areas Driven by Subsurface Flow Velocity Fields

**DOI:** 10.3390/toxics14100905

**Published:** 2026-10-12

**Authors:** Chen Zhao, Qiong Li, Ying Zhang

**Affiliations:** School of Earthquake Engineering and Building Safety, University of Emergency Management, Langfang 065201, China; zhaochen000827@163.com (C.Z.); zhangying830102@126.com (Y.Z.)

**Keywords:** chemical industrial park, groundwater pollution transport, MODFLOW, ammonia nitrogen, 1,2-dichloroethane, subsurface flow velocity

## Abstract

Groundwater pollution in chemical industrial parks is typically concealed, delayed in onset, and difficult to remediate. Moreover, the long-term migration of typical pollutants in aquifers may threaten the safety of surrounding water sources and river water environments. In this study, a numerical subsurface flow model was constructed for a chemical industrial park in a mining area of Inner Mongolia by generalizing the Quaternary loose-rock pore aquifer system, based on hydrogeological surveys and groundwater monitoring data, using MODFLOW. A solute transport model was then established through coupling with MT3DMS to predict the spatiotemporal evolution of ammonia nitrogen and 1,2-dichloroethane in the absence of new pollution sources, and the influence of subsurface flow velocity on pollutant diffusion was further analyzed. The results show that the contaminant plume area gradually increases over the simulation period: the ammonia nitrogen plume expands from 1.84 km^2^ to 3.98 km^2^, and the 1,2-dichloroethane plume expands from 1.47 km^2^ to 3.05 km^2^. Contaminant concentrations in individual wells generally exhibit a downward trend; nonetheless, long-term exceedance risks persist at specific sites. These findings provide a scientific basis for groundwater pollution risk management, water source protection, and subsequent remediation decisions for chemical industrial parks in arid and semi-arid regions.

## 1. Introduction

As China’s industrialization progresses, industrial parks in mining regions have emerged as key bases for resource extraction and processing, and groundwater environmental safety within these parks has attracted increasing attention. Water supply, drainage, and industrial material pipeline networks within these parks were mostly constructed during the early development phases. Affected by prolonged operation, corrosion, aging, and uneven foundation settlement, pipeline leakage has become a severe issue and is now one of the major sources of concealed groundwater contamination in industrial parks [1,2]. Prior research has demonstrated that pipeline leakage continuously releases contaminants and exerts long-term impacts on groundwater quality. Over time, the contaminated area progressively expands, contaminant concentrations rise markedly, and the composite contaminant plume exhibits growing spatial heterogeneity and evolutionary complexity [3,4]. Elevated ammonia nitrogen concentrations can trigger eutrophication in surface water; in groundwater, ammonia nitrogen is transported over long distances and exerts far-reaching influence, making it prone to long-term cumulative pollution [5,6]. 1,2-Dichloroethane is a highly toxic chemical widely used as a precursor in chemical synthesis and as an industrial solvent. Owing to its high density, it readily migrates downward into groundwater, thereby posing significant environmental and health risks [7]. Once aging pipelines leak, contaminants continuously disperse with the subsurface flow to form large-scale pollution plumes, posing a long-term threat to regional drinking-water safety and ecosystems [8,9]. Accordingly, systematically elucidating and quantitatively characterizing the inherent transport behaviors of typical groundwater contaminants in mining-area industrial parks is of considerable practical significance for implementing site-specific groundwater pollution prevention and control schemes, as well as for establishing proactive early-warning systems for pipeline leakage risks.

Numerical simulation has been widely recognized as a vital technical tool for investigating groundwater contaminant migration. Groundwater modeling research emphasizes four key aspects: the reliability of the conceptual model, parameter calibration, uncertainty analysis, and the support for management decision-making derived from model outputs [10,11]. Internationally, the MODFLOW series remains one of the most widely applied finite-difference frameworks for groundwater flow simulation. Continuous advances in research and software tools have given rise to new-generation modeling and automated processing systems, including MODFLOW 6 and FloPy [12,13,14]. MT3DMS and its coupled frameworks are widely used to simulate contaminant advection, dispersion, and reactive processes, and reactive solute-transport models have progressively enhanced their capability to characterize geochemical reactions and multi-component processes [15,16,17]. A large body of research has addressed volatile chlorinated hydrocarbons, DNAPL/VOC plumes, and nitrogen-containing pollutants such as ammonia nitrogen and nitrate, laying a systematic foundation for understanding their migration–transformation behaviors, natural attenuation processes, and associated remediation technologies [18,19,20,21,22,23,24]. Using a reactive transport model, Liang et al. confirmed that rapid rainy-season infiltration inhibits nitrification and promotes ammonia nitrogen accumulation within deep anaerobic aquifers, indicating that neglecting seasonal dynamics could substantially underestimate ammonia nitrogen concentrations in shallow groundwater; this provides a new theoretical basis for the time-dimensional risk assessment of ammonia nitrogen pollution in humid regions [25]. From the perspective of practical field-scale applications, Lu et al. developed a hybrid machine learning–Kriging prediction model that improved the accuracy of exceedance-risk zoning to 0.959 and provided a novel technical pathway for large-scale rapid pollution screening [26]. Using the FEFLOW model, Du et al. meticulously characterized differences in ammonia nitrogen dispersion under various geomembrane defect patterns. Their results indicated that linear cracks tend to produce “tongue-shaped” concentrated contaminant plumes, single-point holes yield the maximum migration distance (up to 188.9 m), and multiple grid-shaped defects generate the largest contaminated area, providing a quantitative basis for landfill-leakage source tracing and risk classification [27].

Huang et al. confirmed via laboratory experiments and saturated-aquifer numerical models that soil-borne fulvic and humic acids significantly alter the adsorption–desorption behavior of 1,2-dichloroethane (1,2-DCA): solid-phase organic matter strengthens adsorption–retardation effects, whereas dissolved liquid-phase organic matter weakens adsorption and facilitates pollutant transport. These findings furnish a mechanistic interpretation for heterogeneous contaminant-plume diffusion within heterogeneous aquifers [28]. Focusing on thick-bed aquifers in southern China, Xie et al. adopted the TMVOC model to simulate the long-term transport behaviors of four DNAPLs, including 1,2-dichloroethane. Their simulation results showed that these pollutants attained a maximum vertical upward migration of 16.90 m and a downstream horizontal migration of 337.77 m within 70 years, representing the highest migration potential among analogous contaminants. Solubility, dispersivity, and hydraulic conductivity were identified as the three major controlling factors for the diffusion ranges. This work filled the knowledge gap regarding DNAPL migration parameters for thick-bed aquifers [29]. Yan et al. compared three remediation strategies–monitored natural attenuation, chemical oxidation, and integrated combined remediation–and revealed that chemical oxidation achieves over 95% pollutant removal within 60 days, while the combined oxidation–natural-attenuation scheme delivers superior long-term control costs, providing quantitative evidence for site-scale remediation-technology screening [30]. Using the GMS platform, Qiu et al. performed long-term numerical predictions and identified the inverse evolutionary relationship between contaminant-plume concentration attenuation and areal expansion; their results can support risk management under no-additional-contaminant-source scenarios [31]. Gao et al. carried out two-dimensional sand-tank visualization experiments and verified that subsurface flow velocity modulates the spatial patterns of DNAPL contaminant plumes: elevated flow velocity substantially enlarges longitudinal migration distances and suppresses lateral diffusion, reshaping plumes from near-circular shapes into narrow elongated strips. This outcome offers theoretical support for contaminant-front prediction and risk management at chlorinated-hydrocarbon-polluted sites [32]. Dumitrescu et al. drew consistent conclusions from regional investigations of plain porous aquifers formed by unconsolidated deposits: the spatial heterogeneity of subsurface flow velocity and hydraulic conductivity constitutes a key hydrological driver governing plume expansion rates and spatial configurations, and higher flow velocity amplifies advection, accelerates longitudinal plume propagation, and extends downstream influence boundaries [33]. Based on three-dimensional numerical simulations for unconfined aquifers, Wang et al. verified that seepage velocity acts as a major controlling factor for benzene-group contaminant-plume evolution: higher seepage velocities shorten the time required to reach plume steady-state conditions, and under strong hydrodynamic regimes, natural attenuation alone cannot achieve effective pollutant abatement. This conclusion supports the selection of natural-attenuation remediation schemes for groundwater-polluted sites [34]. Li et al. conducted river-sand column breakthrough experiments and confirmed that subsurface flow velocity and aquifer grain size jointly govern arsenic-migration retardation: increasing flow velocity notably shortens pollutant breakthrough time and lowers retardation coefficients, and solute transport within coarse-grained aquifers exhibits higher sensitivity to velocity changes. These experimental observations provide technical support for the risk assessment of arsenic migration in heavy-metal-polluted groundwater sites [35].

Methodological frameworks for predicting groundwater contamination in industrial parks have been well established by domestic and international studies. Nevertheless, most previous research has focused primarily on single contaminants or individual contaminated sites. To date, few studies have treated ammonia nitrogen and 1,2-dichloroethane as co-contaminants and carried out numerical simulations of groundwater systems for industrial parks in mining-affected regions using comprehensive hydrogeological survey datasets. Systematic investigations of contaminant migration dynamics in Quaternary porous phreatic aquifers within unconsolidated sedimentary strata across the arid and semi-arid regions of Inner Mongolia remain insufficient. Furthermore, existing research findings fail to fully support refined risk management for river-adjacent water source sites.

To this end, an industrial park located in a mining-affected region of Inner Mongolia was selected as the study area. Based on systematic hydrogeological investigations and groundwater environmental monitoring data, this study generalizes the Quaternary porous unconfined aquifer in unconsolidated sedimentary strata. A three-dimensional groundwater flow model was constructed using MODFLOW and coupled with the MT3DMS module to establish a contaminant transport model. Ammonia nitrogen and 1,2-dichloroethane were selected as the target pollutants to simulate their natural attenuation processes under a scenario with no additional pollution sources, with particular attention paid to their spatiotemporal evolution characteristics and the influence of subsurface flow velocity on pollutant diffusion. Groundwater-level monitoring data collected in December 2025 were used for model calibration and validation. Under the no-additional-pollution-source scenario, this study simulated and predicted the spatiotemporal evolution of both pollutants at four time points (30 days, 1 year, 5 years, and 10 years) to assess the potential risks to the surrounding water environment. The results quantitatively reveal the migration distances, diffusion extents, and concentration attenuation patterns of ammonia nitrogen and 1,2-dichloroethane in the unconsolidated porous aquifer and clarify their exceedance zones and impact boundaries across different prediction periods. The study results provide a solid scientific basis for groundwater pollution risk assessment, early warning of aging-pipeline leakage, and optimization of groundwater pollution prevention and control schemes in mining-affected industrial parks.

## 2. Model Construction and Validation

### 2.1. Simulated Area

The study area is an industrial park in Inner Mongolia with a planned area of 25.9 km^2^. As shown in Figure 1, the region features a continental arid climate with low precipitation, frequent winds, and high evaporation. The terrain tilts from west to east at a gradient of approximately 3‰, with minor north-to-south undulations, presenting an overall pattern of higher elevation in the south and lower elevation in the north. Surface elevations range from 1120 m to 1320 m, and the site terrain is virtually flat without pronounced relief. Hydrogeologically, the study area lies within a Quaternary unconsolidated porous unconfined aquifer system comprising Holocene and Upper Pleistocene alluvial–diluvial sand–gravel and cobble deposits. Lacking stable aquitards, these strata form a unified, exceptionally thick unconfined aquifer system. Groundwater is recharged primarily by atmospheric precipitation infiltration and lateral seepage from the Yellow River, whereas regional discharge occurs predominantly via lateral runoff toward the Yellow River.

### 2.2. Hydrogeological Conceptual Model and Grid Discretization

The simulated area covers approximately 46.89 km^2^. The western mountainous terrain was defined as a specified-flux boundary, and the eastern Yellow River was designated as a constant-head boundary. The northern and southern boundaries were set as no-flow boundaries, making the study area a closed and independent hydrogeological unit. The subsurface flow model was constructed using MODFLOW, with a spatial discretization of 58 rows by 56 columns, yielding 2905 active cells. Local grid refinement was applied to key areas of interest. Based on the formulation adopted by Wang et al. [36], contaminants are treated as particles advected by water flow, and their migration patterns are simulated by tracking the trajectories of these particles.

### 2.3. Key Parameter Settings

#### 2.3.1. Basic Parameter Settings

Based on the analysis of hydrogeological conditions, topographical features, groundwater flow fields, and calculation results from field pumping tests, the aquifer of the study area was divided into five zones with different hydraulic conductivity values (Figure 2).

According to the requirements of the hydrogeological mathematical model, the hydrogeological parameters and source–sink terms for the calibration period are listed in Table 1 and Table 2, including hydraulic conductivity (K), specific yield (μ), and longitudinal dispersivity (α_L_). Recharge comprised precipitation infiltration and lateral groundwater inflow, whereas discharge consisted of lateral groundwater outflow. The groundwater table depth in this region ranges from 72 m to 142 m, which exceeds the evaporation extinction depth; therefore, evaporation was neglected in the model. Furthermore, contaminant-related processes, including sorption, ion exchange, volatilization, and biochemical reactions, were excluded from the simulation. The data presented in Figure 2 and Table 1 are derived from in situ field measurements carried out within our commissioned project.

#### 2.3.2. Initial Concentration of Groundwater Contamination

Compared with the preselected initial concentration dataset from December 2025, only two monitoring points (W13 and W14) were newly added to the water-quality monitoring network of 14 shallow groundwater wells in the study area in April 2026. Accordingly, the initial concentrations were revised based on the monitoring data obtained in April 2026. Water-quality monitoring results from the 14 shallow groundwater wells in April 2026 indicated that five parameters exceeded the Class III limits specified in the Standard for Groundwater Quality [37]: ammonia nitrogen (NH3-N), vinyl chloride (VC), trichloroethylene (TCE), 1,1,2-trichloroethane (1,1,2-TCA), and 1,2-dichloroethane (1,2-DCA).

As shown in Figure 3, ammonia nitrogen (NH3-N) reached a maximum concentration of 69.3 mg/L, exceeding its regulatory limit (0.5 mg/L) by a factor of 138.6. The maximum concentration of 1,2-dichloroethane (1,2-DCA) was 6.27 mg/L, representing a 209-fold exceedance of its limit of 0.03 mg/L. Vinyl chloride (VC), trichloroethylene (TCE), and 1,1,2-trichloroethane (1,1,2-TCA) reached maximum concentrations of 0.233 mg/L (46.6-fold exceedance), 0.283 mg/L (4.04-fold exceedance), and 0.225 mg/L (45-fold exceedance), respectively. Ammonia nitrogen and 1,2-DCA exhibited the highest exceedance ratios among the five pollutants and were therefore selected as the target pollutants for subsequent analysis.

### 2.4. Model Calibration and Validation

The initial simulation timestamp (t = 0) was set to December 2025, which was defined as the baseline period for model calibration. Manual calibration was performed by iteratively adjusting key hydrogeological parameters, with borehole water-level monitoring data from December 2025 adopted as the calibration benchmark. The simulated and measured water levels showed minimal deviation, and the simulated groundwater-level contour lines (Figure 4) were consistent with the observed patterns. As shown in Figure 5, linear regression between the simulated and observed hydraulic heads yielded an R^2^ of 0.97444 with a slope of 0.99, and all monitoring points fell within the 95% prediction interval. The simulated and observed hydraulic heads exhibited good consistency. This strong agreement demonstrates that the model accurately captures groundwater dynamics in the study area and that the selected hydrogeological parameters are reasonable.

## 3. Results

### 3.1. Prediction Results

#### 3.1.1. Analysis of Ammonia Nitrogen Pollution Prediction Results

The transport and dispersion characteristics of ammonia nitrogen (NH3-N) in groundwater across different simulation periods are illustrated in Figure 6. The migration of ammonia nitrogen within groundwater systems is governed jointly by hydrodynamic dispersion, recharge conditions, redox environments, and aquifer media properties.

The initial concentration of NH3-N exceeded the regulatory limit by a factor of 138.6. After 30 days of simulation, the maximum NH3-N concentration was 66.77 mg/L (133.54 times the 0.5 mg/L Class III groundwater limit), and the contaminant plume began to migrate with the subsurface flow toward the Yellow River. After one year, the plume advanced further toward the Yellow River, with the maximum concentration reaching 43.32 mg/L (86.64 times the limit). After five years, the maximum concentration dropped to 26.66 mg/L (53.32 times the limit). By year 10, the maximum concentration had decreased to 20.21 mg/L (40.42 times the limit). Although concentrations at individual locations decreased by the end of the simulation period, the contaminant still exerts a sustained impact on the surrounding groundwater. Furthermore, a water source area situated between the industrial park and the Yellow River requires priority protection. Detailed predictive simulation results for contaminant transport are presented in Figure 7.

#### 3.1.2. Analysis of 1,2-Dichloroethane Pollution Prediction Results

Figure 8 illustrates the transport and dispersion of 1,2-dichloroethane (1,2-DCA) in groundwater over different simulation periods. Volatile halogenated hydrocarbons and chlorinated solvents typically exhibit high mobility and pose long-term residual risks. The evolution of the 1,2-DCA contaminant plume is influenced by the source-zone release history, aquifer heterogeneity, and natural attenuation processes.

The initial 1,2-DCA concentration exceeded the standard limit by a factor of 209. After 30 days of simulation, the maximum 1,2-DCA concentration was 6104.13 μg/L (203.47 times the 30 μg/L Class III groundwater limit), and the contaminant plume began to migrate in the direction of the subsurface flow toward the Yellow River. After one year, the plume advanced further toward the Yellow River, reaching a maximum concentration of 4438.54 μg/L (147.95 times the limit). After five years, the maximum concentration dropped to 2561.15 μg/L (85.37 times the limit). By year 10, the maximum concentration had decreased to 1757.49 μg/L (58.58 times the limit). Although 1,2-DCA concentrations at individual locations decreased by the end of the simulation period, the contaminant still exerts a sustained impact on the surrounding groundwater. Furthermore, a water source area situated between the industrial park and the Yellow River requires priority protection. Detailed predictive simulation results are presented in Figure 6.

As illustrated in Figure 6, the exceedance areas and maximum transport distances of both contaminants exhibit a continuously increasing trend as the prediction period extends from 30 to 3600 days. Specifically, the exceedance areas of ammonia nitrogen (NH3-N) and 1,2-dichloroethane (1,2-DCA) expanded by 2.14 km^2^ and 1.58 km^2^, respectively. Across all prediction time points, the exceedance area of NH3-N remains consistently larger than that of 1,2-DCA, indicating that NH3-N possesses a stronger longitudinal dispersion capacity within the aquifer and is more prone to forming a broader contaminant plume, thereby exerting a more extensive impact on the regional groundwater environment.

Over the prediction period from 30 to 3600 days, the contaminant plumes of ammonia nitrogen (NH3-N) and 1,2-dichloroethane (1,2-DCA) migrated laterally toward the Yellow River by 2193 m and 1975 m, respectively. In the initial stage of the prediction, the maximum transport distance of 1,2-DCA was slightly greater than that of NH3-N, indicating a faster lateral migration rate. However, as the prediction time extended beyond 1800 days, the lateral transport distance of NH3-N gradually surpassed that of 1,2-DCA, and their maximum transport distances ultimately converged.

Without the implementation of control measures, there is a significant long-term risk of groundwater contamination. These findings provide a scientific basis for formulating differentiated groundwater pollution prevention and control strategies in the study area: regional containment strategies should be prioritized for NH3-N, while measures to intercept the downstream contaminant front should be strengthened for 1,2-DCA.

### 3.2. Effects of Subsurface Flow Velocity on Contaminant Diffusion Characteristics

To evaluate the simulation results and investigate the effects of longitudinal and transverse flow velocities on the transport and diffusion of groundwater pollutants, a grid of observation points (11 rows by 14 columns) was established around the monitoring wells depicted in Figure 1.

#### 3.2.1. Impact of Longitudinal Flow Velocity on Diffusion

Figure 9 illustrates the spatial distributions of pollutant concentrations and longitudinal subsurface flow velocities over simulation periods of 30, 360, 1800, and 3600 days. The monitoring cross-section, extending from G8-1 to G8-11, corresponds sequentially to the flow direction from upstream to downstream. The left vertical axis represents the subsurface flow velocity in the x-direction (m/d), while the right vertical axis denotes the pollutant mass concentration in groundwater (mg/L). Based on the simulation results, the following insights can be drawn:

(1) Characteristics of Longitudinal Seepage Velocity

During each simulation period, the longitudinal groundwater seepage velocity along the flow direction (from G8-1 to G8-11) exhibited a linear decreasing trend from upstream to downstream, indicating that the hydraulic driving force along the flow path gradually weakens, resulting in a continuous decline in groundwater runoff intensity. As illustrated in Figure 6 and Figure 7, the pollutants migrated eastward with the subsurface flow. Over time, the volume of lateral recharge and the corresponding lateral flow velocities increased, which drove the progressive expansion of pollutant dispersion along the flow direction, extended the eastward migration distance of the contaminant plume, and led to a gradual decrease in pollutant concentrations.

A greater absolute slope value corresponds to a more rapid decrease in flow velocity, causing the zone of enhanced lateral pollutant spreading to occur closer to the upstream source. Driven by the continuous regulation of the subsurface flow field, the absolute value of the slope increases from 0.543 × 10^−4^ at 30 days to a peak of 2.32 × 10^−4^ at 1800 days; by 3600 days, the absolute slope value decreases to 1.02 × 10^−4^, accompanied by a narrowed spatial gradient, indicating that the subsurface flow field has progressively entered a dynamically stable stage. As illustrated in Figure 6 and Figure 7, this evolution is manifested by the progressive expansion of the contaminant plume front over time.

(2) Contaminant Transport and Attenuation Processes

Although 1,2-DCA and ammonia nitrogen share similar attenuation patterns, they exhibit distinct maximum-concentration locations due to differences in their initial source distributions. The migration and attenuation of both pollutants demonstrate clear phased evolution patterns:

In the early stage (30–360 days), high-concentration pollutants are predominantly accumulated in the upstream region near the source (G8-1 to G8-5). During this period, the ammonia nitrogen concentration at the upstream points ranges from 66.438 to 11.365 mg/L, while the peak 1,2-DCA concentration spans from 3.14 to 0.132 mg/L. Concentrations of both pollutants remain relatively low downstream of G8-8, and the contaminant plumes have not yet migrated far downstream.

In the middle stage (1800 days), driven by advection–diffusion and hydraulic forces, the upstream pollutants undergo dilution and degradation, while the contaminant plumes advance significantly downstream (from G8-7 to G8-11), forming secondary concentration peaks (e.g., NH3-N reaching 10.16 mg/L and 1,2-DCA reaching 0.38 mg/L at G8-8).

In the late stage (3600 days), concentrations across the entire cross-section decline sharply, with those in the upper and middle reaches approaching zero; only the furthest downstream reaches (G8-9 to G8-11) retain exceptionally low residual concentrations. This indicates that the system has undergone a relatively thorough process of natural self-purification and migration out of the model domain.

#### 3.2.2. Impact of Transverse Flow Velocity on Diffusion

Figure 10 illustrates the spatial distributions of pollutant concentrations and transverse groundwater flow velocities over simulation periods of 30, 360, 1800, and 3600 days. The monitoring cross-section, extending from G8-1 to G8-11, corresponds sequentially to the flow direction from upstream to downstream. The left vertical axis represents the groundwater transverse flow velocity (m/d), while the right vertical axis denotes the pollutant mass concentration in groundwater (mg/L). Based on the simulation results, the following insights can be drawn:

(1) Characteristics of Transverse Seepage Velocity

The transverse flow velocity of groundwater exhibits a decreasing trend with increasing transport distance, indicating that the hydraulic driving force along the flow path gradually weakens and the runoff intensity continuously declines. As illustrated in Figure 6 and Figure 7, the lateral width of the contaminant plume progressively expands along the flow direction. Over time, the volume of lateral recharge and the corresponding horizontal flow velocities increase, driving the progressive expansion of pollutant dispersion, extending the eastward migration distance of the contaminant plume, and leading to a gradual decrease in pollutant concentrations.

Along the monitoring cross-section (G8-1 to G8-11), the transverse seepage velocity (*V_y_*) exhibits a spatially decreasing pattern, shifting from northward flow (*V_y_* > 0) in the upstream reaches (G8-1 to G8-5) to a gradual southward deflection in the downstream reaches (G8-7 to G8-11), with the coefficients of determination (R^2^) for the 30–1800 day fittings ranging from 0.7756 to 0.8771. In the early stage of the simulation (30–360 days), the hydraulic field exhibits a northward driving force, with the northward velocity peaking at 0.23 m/d upstream by 360 days. As the simulation progresses to the middle stage (1800 days), the northward driving force gradually decays, and *V_y_* across the entire cross-section approaches zero. By the late stage (3600 days), the subsurface flow direction undergoes a complete reversal: except at the starting point, *V_y_* across the entire cross-section drops to between −0.05 and −0.15 m/d, demonstrating a pattern of comprehensive southward flow.

(2) Contaminant Transport and Attenuation Processes

The spatiotemporal distribution of contaminants is significantly regulated by the dynamic evolution of the north–south hydraulic field. In the early stage (30–360 days), the northward velocity component (*V_y_* > 0) effectively confines high-concentration pollutants to the upstream area near the source (G8-1 to G8-5). During this period, the ammonia nitrogen concentration at the upstream points ranges from 66.438 to 11.365 mg/L, while the peak 1,2-DCA concentration spans from 3.14 to 0.132 mg/L. Concentrations of both pollutants remain relatively low downstream of G8-8, and the contaminant plumes have not yet migrated far downstream. In the middle stage (1800 days), as the northward driving force weakens and *V_y_* approaches zero, the contaminant plumes advance smoothly downstream and form secondary peaks near G8-8 (reaching 10.16 mg/L for NH3-N and 0.38 mg/L for 1,2-DCA). In the late stage (3600 days), the subsurface flow direction completely reverses southward (*V_y_* < 0). Driven by natural attenuation and discharge processes, the pollutants in the upper and middle reaches are largely eliminated, leaving only an exceptionally low-concentration contaminant plume that slowly migrates toward the southern boundary for dissipation.

## 4. Conclusions

The three-dimensional groundwater flow and coupled solute transport model constructed in this study passed rigorous calibration and validation against the December 2025 field monitoring data, with a coefficient of determination R^2^ = 0.974 and a regression slope of 0.99. All monitored hydraulic head points fell within the 95% prediction interval, confirming that the generalized Quaternary unconsolidated porous phreatic aquifer system and the adopted set of hydrogeological parameters are site-specific and physically consistent. The model can reliably reproduce the regional groundwater seepage field and support long-term prediction of contaminant transport under no-new-pollution-source scenarios.

Under the no-additional-pollution-source scenario, the spatiotemporal evolution of ammonia nitrogen and 1,2-dichloroethane shows distinct differentiation patterns over the 10-year prediction period. The ammonia nitrogen plume grows from the initial 1.84 km^2^ to 3.98 km^2^ by year 10, with an east–west maximum migration distance of 2193 m, and its peak concentration decays from 69.3 mg/L to 20.21 mg/L, dominated by advection–dilution attenuation. The 1,2-dichloroethane plume expands from 1.47 km^2^ to 3.05 km^2^ within 10 years. The long-term prediction reveals that 6 monitoring wells will continuously exceed the GB/T 14848-2017 [37] Class III water quality limit over the 10-year period, and 3 of them will remain at high exceedance levels even after 10 years.

Quantitative parameter sensitivity analysis further confirms that groundwater seepage velocity is the dominant hydrological driver controlling the spatiotemporal configuration of both contaminant plumes in this thick unconfined aquifer. The longitudinal seepage velocity (along the main flow direction toward the Yellow River) ranges from 1.27 to 2.45 m/d, accounting for more than 76% of the total scalar velocity and constituting the dominant driving force for contaminant advection. The transverse seepage velocity perpendicular to the main flow direction is only 0.23 to 0.58 m/d, with an average of 0.37 m/d, far lower than the longitudinal velocity component. This velocity anisotropy directly explains the morphological evolution differences between the two contaminant plumes: under the current velocity field conditions, the longitudinal expansion rate of the ammonia nitrogen plume is 4.2 times its lateral diffusion rate, the 10-year migration distance in the downstream direction reaches 2193 m, and the maximum lateral spread width is only 520 m; the longitudinal expansion rate of the 1,2-dichloroethane plume is 7.3 times its lateral diffusion rate, the 10-year migration distance in the downstream direction reaches 1975 m, and the maximum lateral spread width is only 270 m. These findings confirm that, in the unconsolidated porous aquifers of arid and semi-arid mining areas, advection remains the leading control factor for ammonia nitrogen pollution, whereas the diffusion of 1,2-dichloroethane shows higher sensitivity to the spatial heterogeneity of hydraulic conductivity.

This study did not incorporate the full geochemical reaction network or the seasonal dynamics of groundwater recharge into the current simulation, which may cause a slight overestimation of the 10-year diffusion range of ammonia nitrogen. Future research will focus on the refined prediction of the spatiotemporal distribution of pollutant concentration gradients under multi-scenario rainfall infiltration conditions.

## Figures and Tables

**Figure 1:**
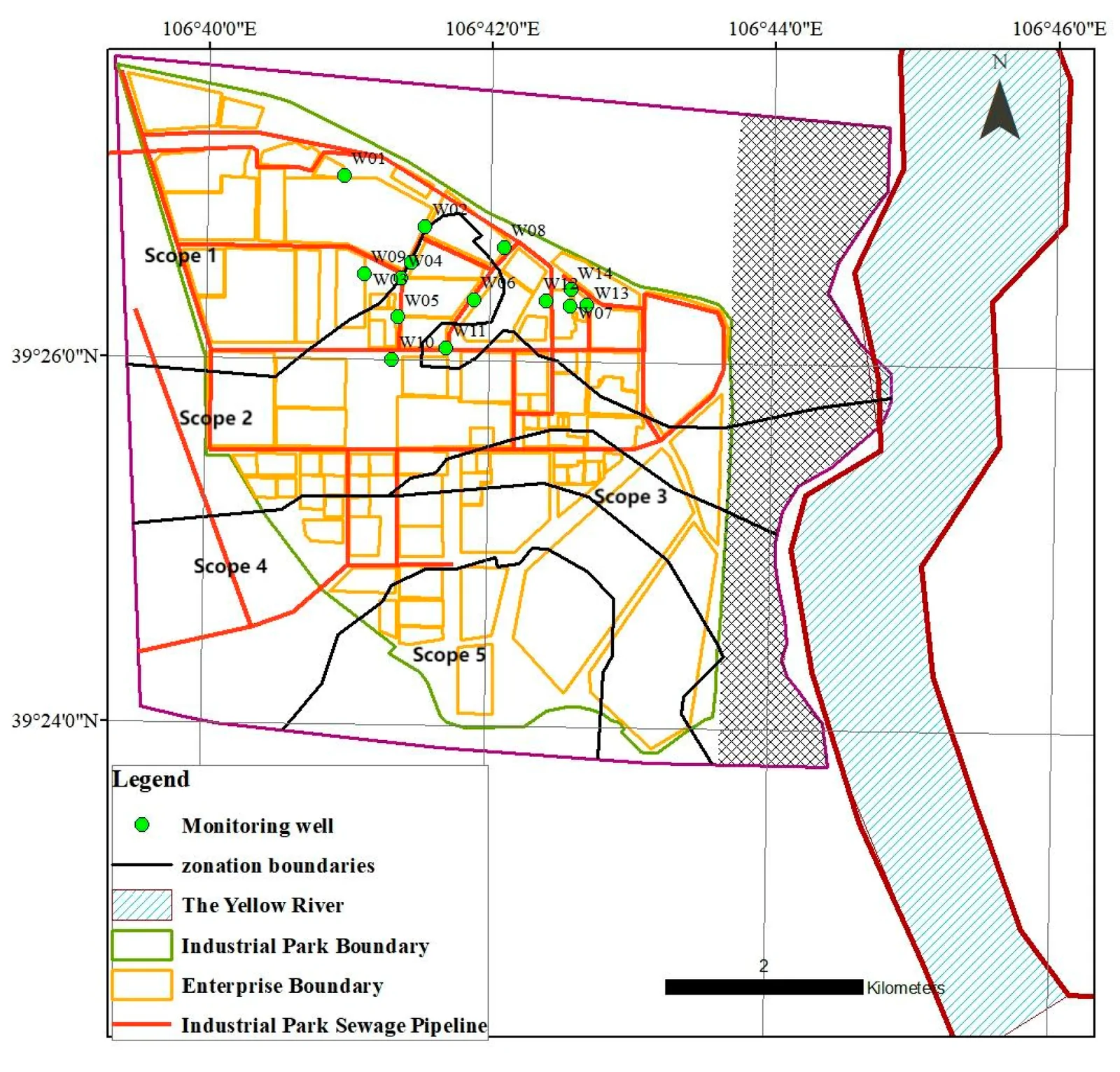
Schematic diagram of the study area and hydrogeological parameter zonation.

**Figure 2:**
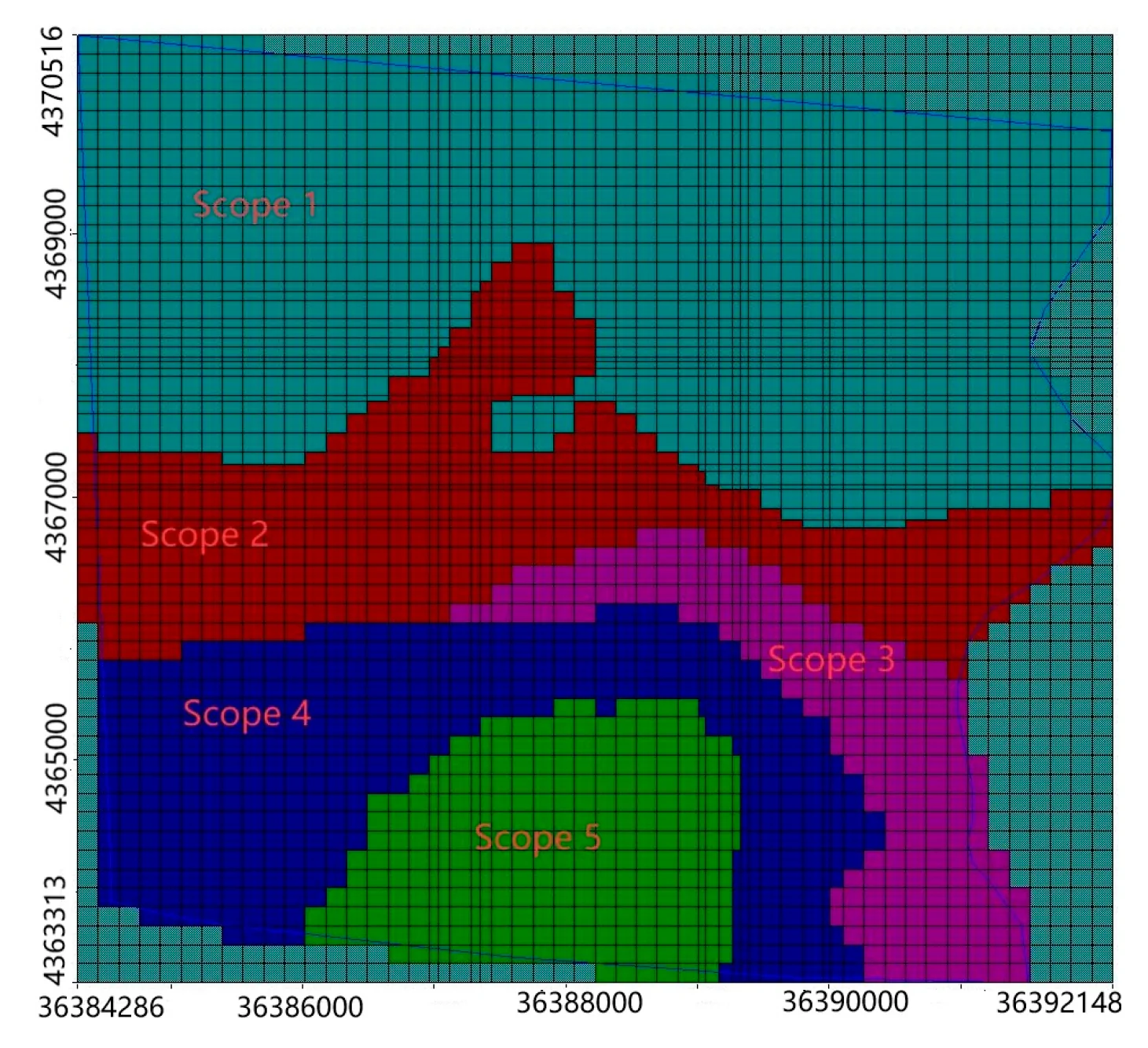
Hydrogeological parameter zonation.

**Figure 3:**
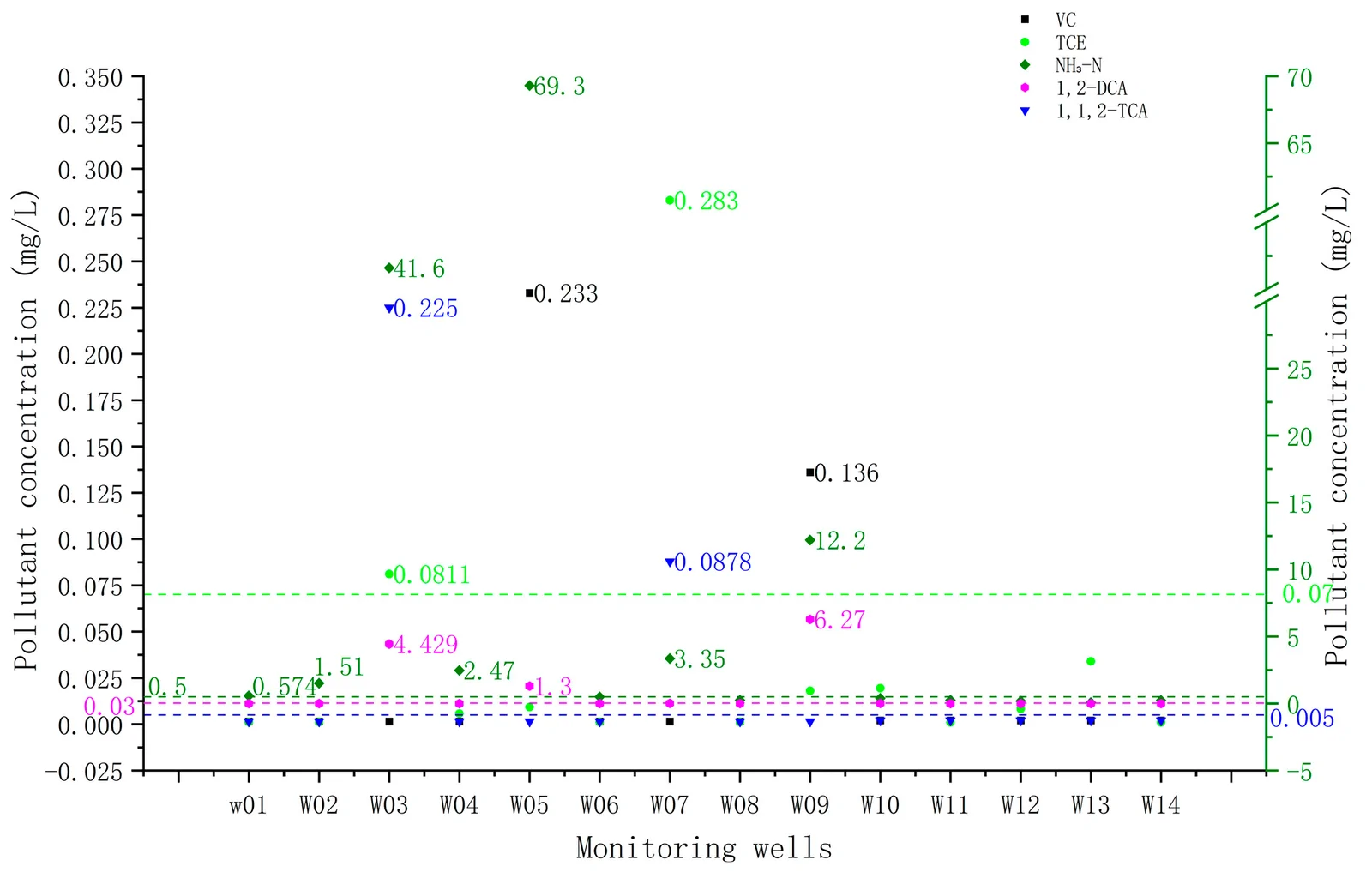
Analysis of parameter exceedances across the monitoring wells.

**Figure 4:**
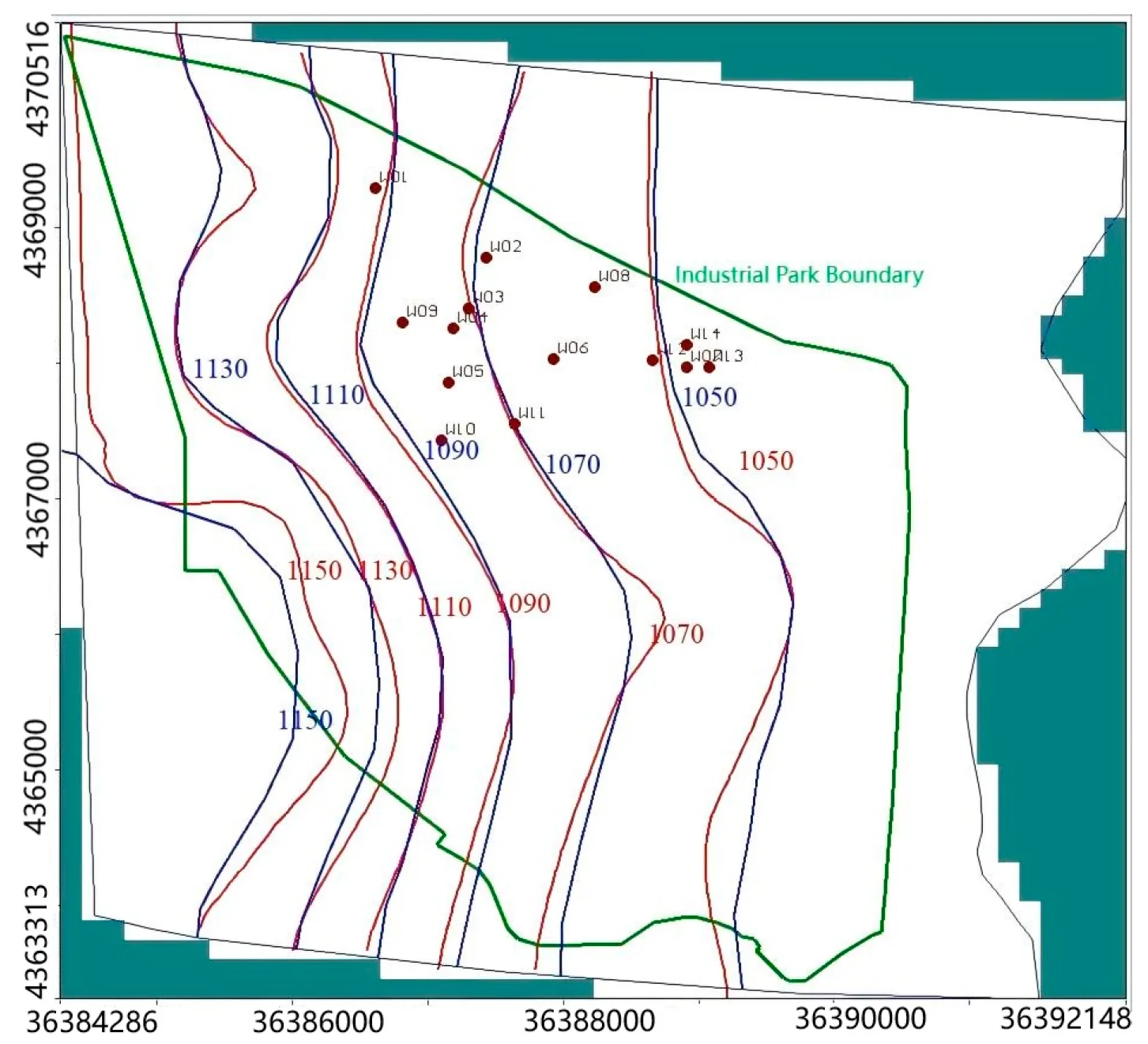
Comparison between simulated and observed groundwater levels (blue lines: observed groundwater contours at the end of the calibration period; red lines: simulated groundwater contours at the end of the calibration period).

**Figure 5:**
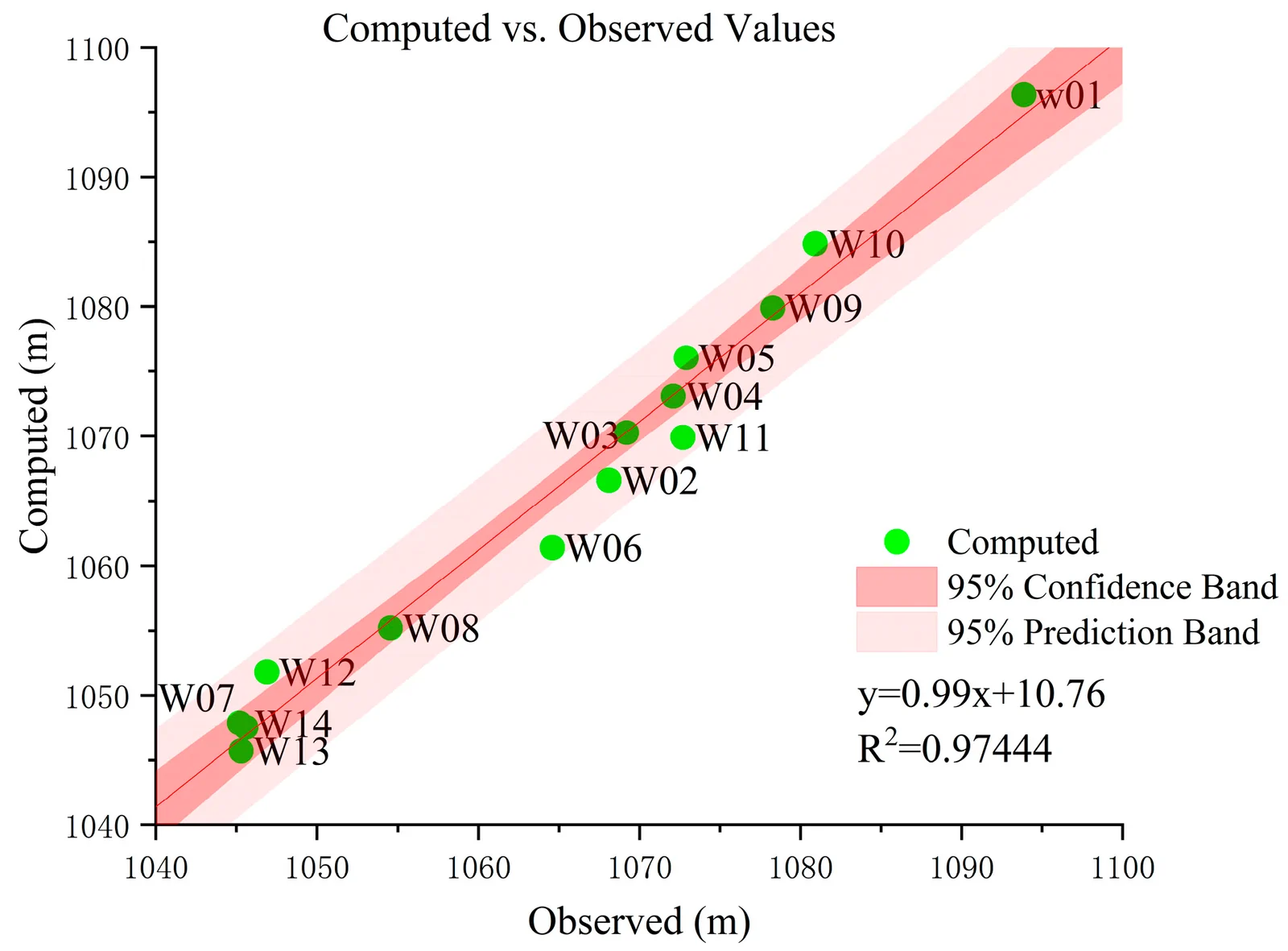
Scatter plot of simulated versus observed groundwater levels at the boreholes.

**Figure 6:**
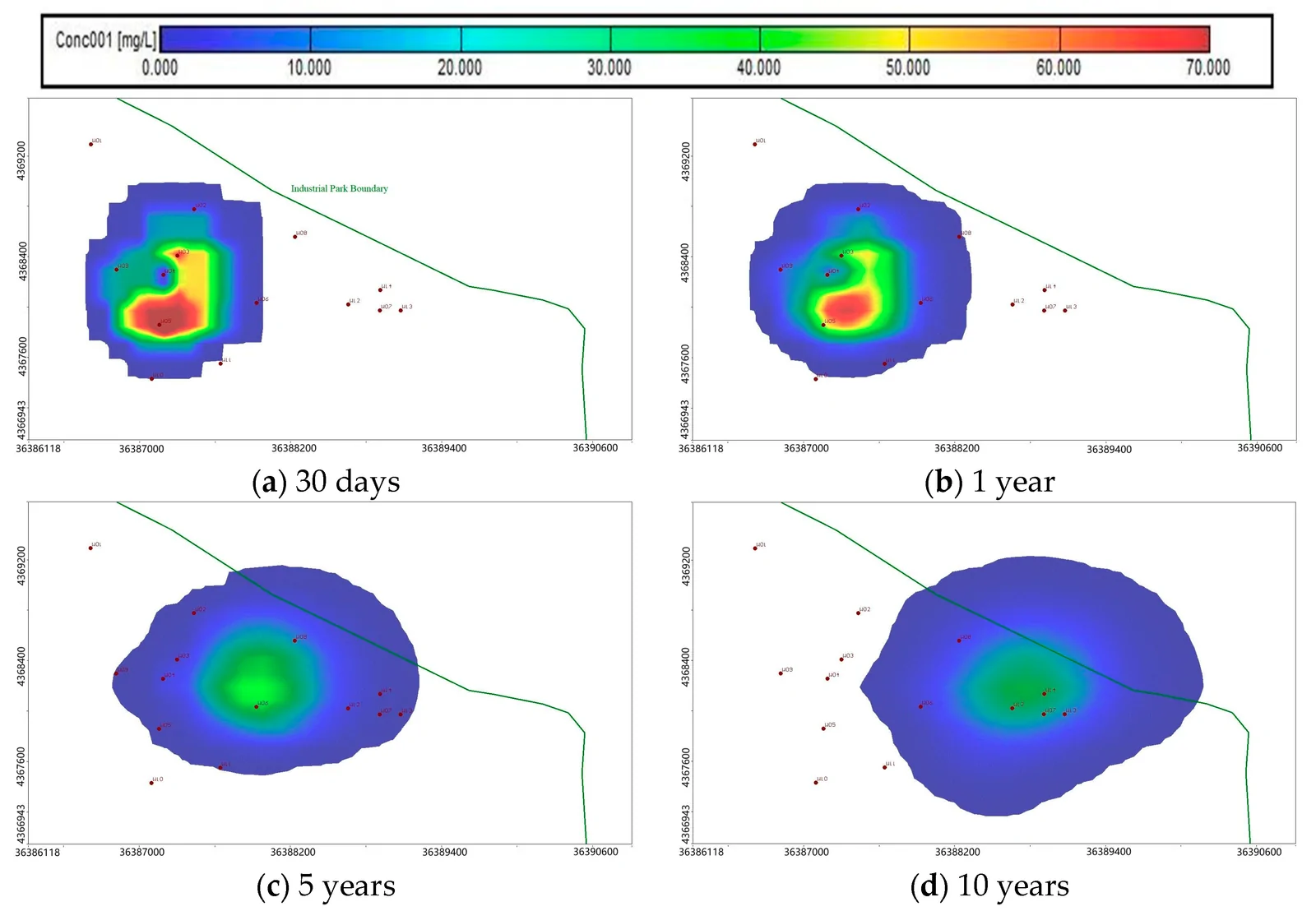
Predicted plume extent of ammonia-nitrogen.

**Figure 7:**
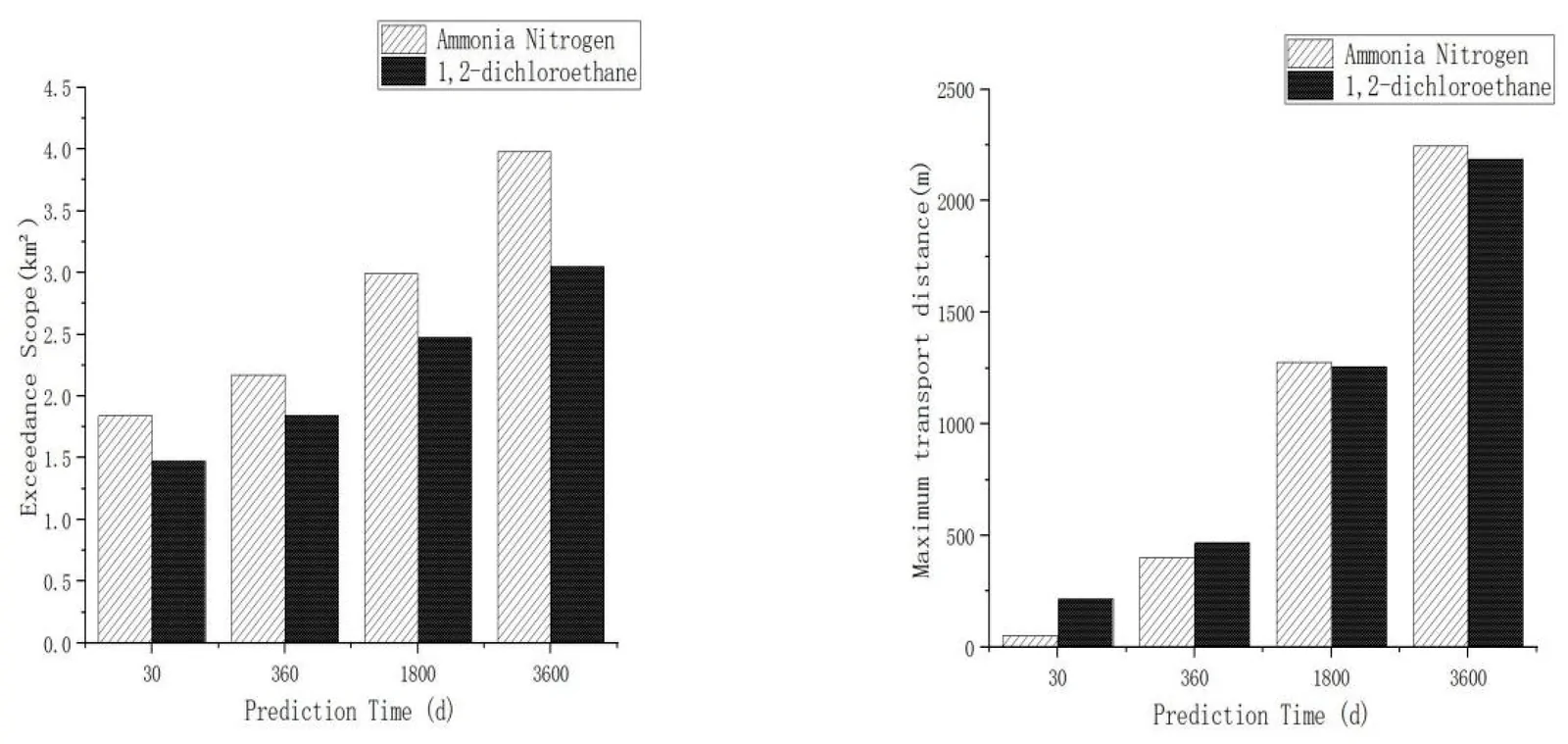
Exceedance extent and maximum migration distance of pollutants under uncontrolled conditions in the industrial park.

**Figure 8:**
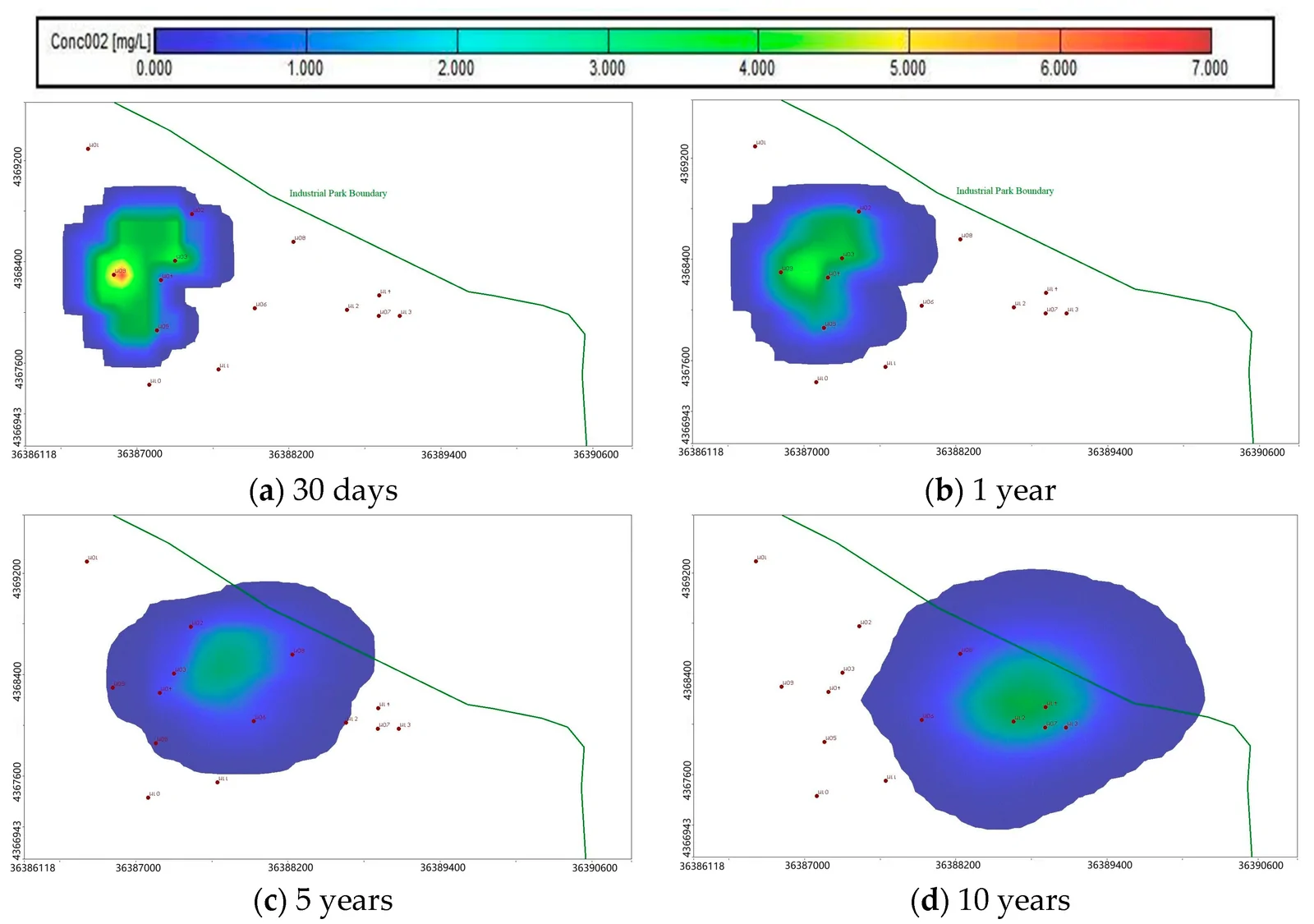
Predicted plume extent of 1,2-dichloroethane.

**Figure 9:**
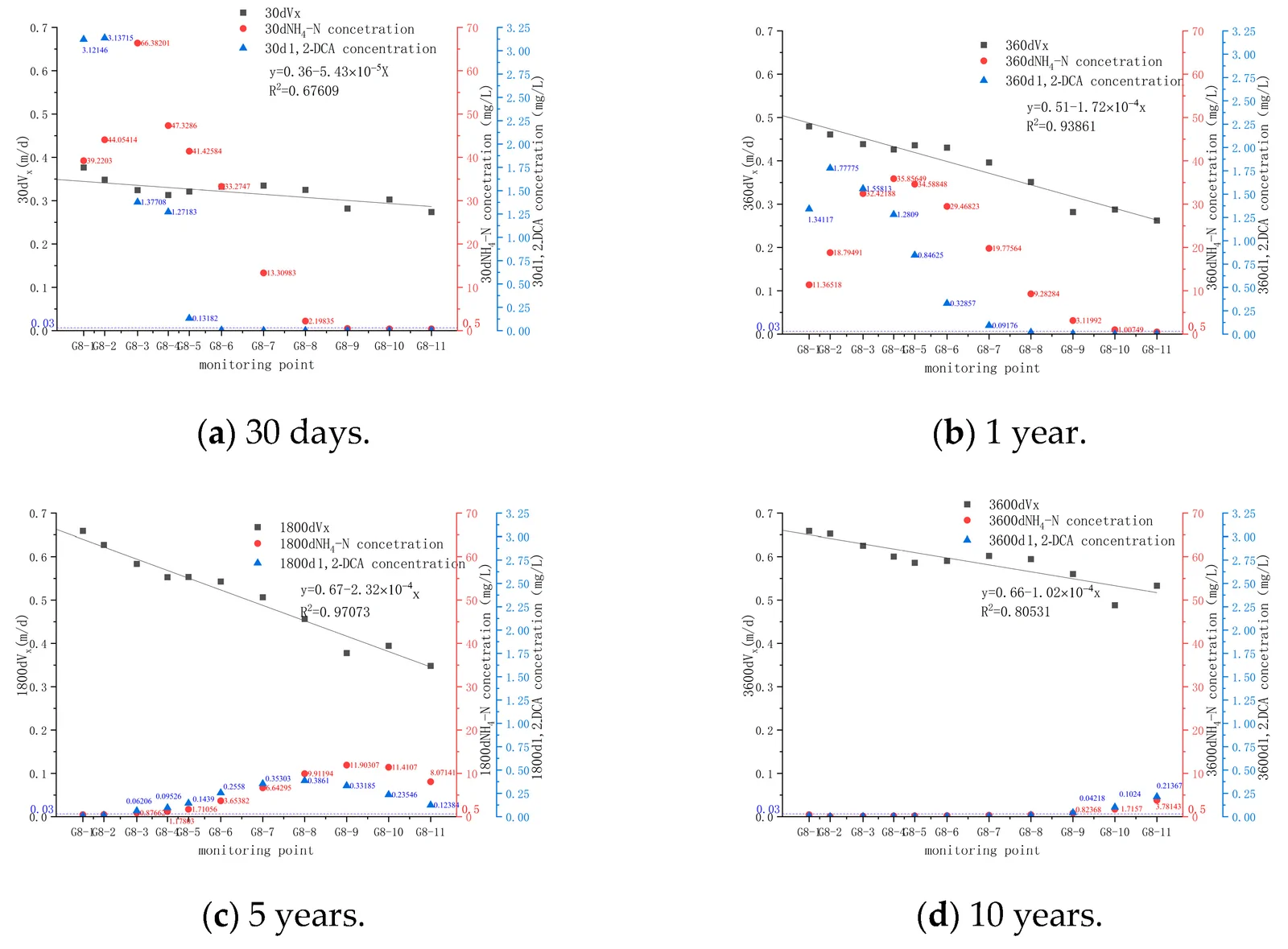
Correlation curves between pollutant concentration and longitudinal subsurface flow velocity.

**Figure 10:**
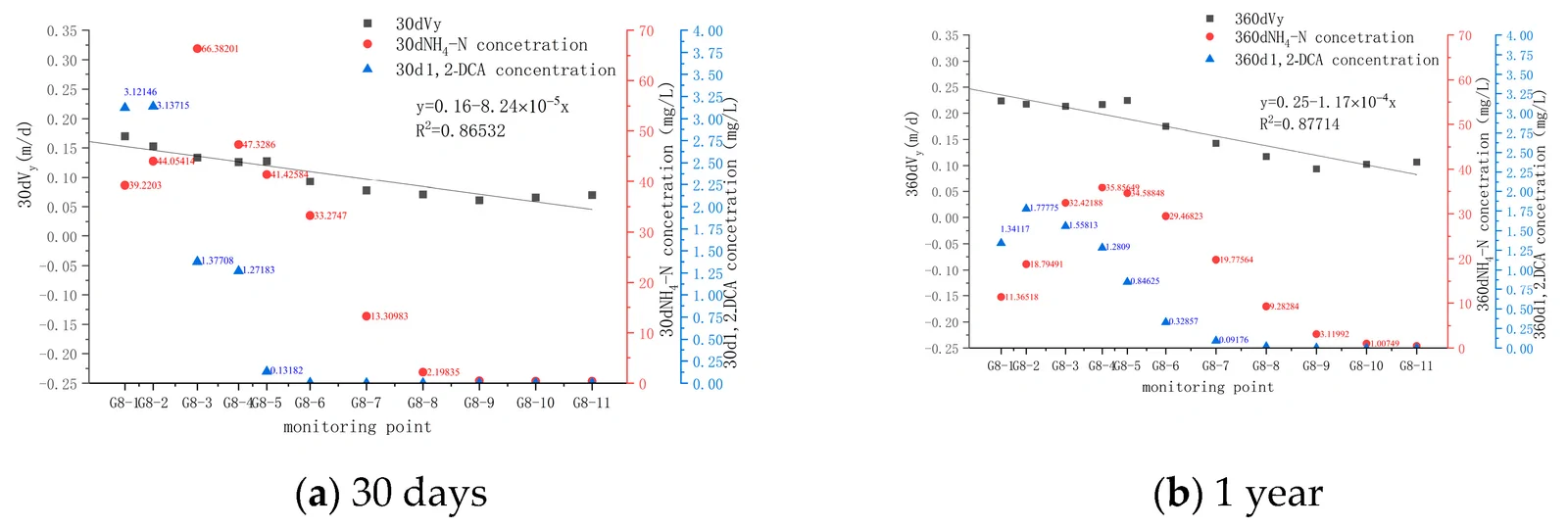

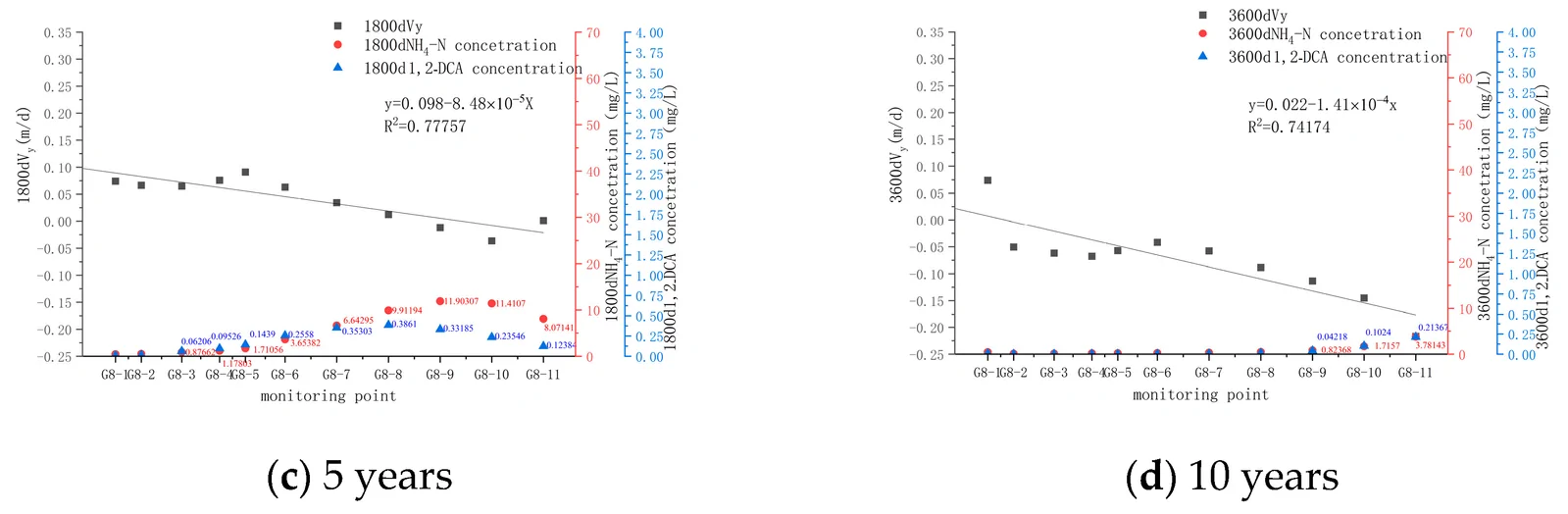
Correlation curves between pollutant concentration and transverse subsurface flow velocity.

**Table 1:** Hydrogeological parameters.

Parameter Zone	Hydraulic Conductivity (K)	Specific Yield of the Unconfined Aquifer (μ)	Longitudinal Dispersivity (αL)
Unit	m/d	Dimensionless	m
1	2.56	0.12	1
2	3.88	0.18	1
3	5.15	0.2	1
4	6.5	0.23	1
5	8.7	0.25	1

**Table 2:** Summary of source/sink terms during the calibration period (Unit: 10^4^ m^3^).

Source/Sink Terms		Volume	Total
Recharge terms	Precipitation infiltration	0.09745	10.36225
	Lateral groundwater inflow	10.2648	
Discharge terms	Lateral groundwater outflow	9.19058	9.19058
Discrepancy			1.17167

## Data Availability

The data presented in this study are available on request from the corresponding author due to the restrictions of the commissioned project.
